# Analysis of the Efficacy of Universal Screening of Coronavirus Disease with Antigen-Detecting Rapid Diagnostic Tests at Point-or-Care Settings and Sharing the Experience of Admission Protocol—A Pilot Study

**DOI:** 10.3390/jpm12020319

**Published:** 2022-02-20

**Authors:** Ji Young Park, Joo Hee Lee, Bong Ki Cha, Boo-Seop Kim, Han-Jun Lee, Gi Hyeon Kim, Kyu-Tae Kang, Yong-Soon Lee, Seok Keun Ahn, Seong Hwan Kim

**Affiliations:** 1 Department of Pediatrics, Chung-Ang University Hospital, 102, Heukseok-ro, Dongjak-gu, Seoul 06973, Korea; jypark@caumc.or.kr; 2 Department of Internal Medicine, Hyundae General Hospital, Chung-Ang University, Gyunggi-do, Namyangju-si 12013, Korea; hd20190205@hdgh.co.kr (J.H.L.); vonga9577@gmail.com (B.K.C.); 3 Department of Orthopaedic Surgery, Hyundae General Hospital, Chung-Ang University, Gyunggi-do, Namyangju-si 12013, Korea; ybkkbs@gmail.com (B.-S.K.); mnlwhexo2@moakt.ws (Y.-S.L.); 4 Department of Orthopaedic Surgery, Chung-Ang University Hospital, 102, Heukseok-ro, Dongjak-gu, Seoul 06973, Korea; gustinolhj@nate.com (H.-J.L.); aldjfh123@caumc.or.kr (K.-T.K.); 5 Department of Radiology, Hyundae General Hospital, Chung-Ang University, Gyunggi-do, Namyangju-si 12013, Korea; ds6gmy3nu@tmpbox.net; 6 Department of Emergency Medicine, Hyundae General Hospital, Chung-Ang University, Gyunggi-do, Namyangju-si 12013, Korea; ahnseokkeun@gmail.com

**Keywords:** COVID-19, cross infection, infection control, SARS-CoV-2

## Abstract

Aims: To introduce the admission protocol of a COVID-19 specialized hospital outlined by the government, including the assessment of reverse transcription polymerase chain reaction (RT-PCR), low dose chest computed tomography (CT) and antigen-detecting rapid diagnostic test (Ag-RDT) for patient screening. Materials and Methods: This was a retrospective cohort study of 646 patients who were admitted between December 2020, and February 2021, during the third wave of COVID-19 in Korea. Ag-RDT and RT-PCR were routinely performed on all patients who required admission, and low-dose chest CT was performed on high-risk patients with associated symptoms. Any patients with high-risk COVID-19 infection according to the Ag-RDT test were quarantined alone in a negative pressured room, and those with low-risk COVID-19 infection remained in the preemptive quarantine room with or without negative pressure. The diagnostic values of the Ag-RDT test and associated cycle threshold (Ct) values of the RT-PCR test were subsequently evaluated. Results: In terms of the diagnostic value, the Ag-RDT for COVID-19 had a sensitivity of 68.3%, specificity of 99.5%, positive predictive value (PPV) of 90.3%, and negative predictive value (NPV) of 97.9%. For the 355 symptomatic patients with low-dose chest CT, the diagnostic values of combined evaluations had a sensitivity of 90.2%, specificity of 99.0%, PPV of 86.1%, and NPV of 99.3%. The cut-off Ct value for positive Ag-RDT was ≤25.67 for the N gene (sensitivity: 89.3%, specificity: 100%), which was regarded as a high viable virus in cell culture. There were no patients or medical staff who had COVID-19 in the hospital. Conclusion: Appropriate patient care was possible by definitive triage of the area, according to the symptoms and using diagnostic tests. Screening protocols, including the Ag-RDT test and low-dose chest CT, could be helpful in emergency point-of-care settings.

## 1. Introduction

Coronavirus disease 2019 (COVID-19) has been spreading across the globe, undergoing mutational evolution [1,2]. In Korea, the first COVID-19 patient was diagnosed in January of 2020 [3]; since then, the number of patients has steadily increased throughout three outbreaks over one year [4]. Although typical symptoms of COVID-19 are fever and respiratory symptoms, this disease is asymptomatic in most young people [5,6,7]. Among patients with other symptoms or underlying diseases, some of them were diagnosed with COVID-19 by reverse transcription polymerase chain reaction (RT-PCR) before admission or operation. Thus, this can complicate the procedure for clinicians during the examination and treatment of non-COVID-19 patients at outpatient or emergency departments.

Many research articles have focused on the association between cycle threshold (Ct) values in RT-PCR and viral cultures [8,9,10,11,12]. Identifying viable viruses through viral culture is difficult in clinical settings as specimen handling must be performed in biosafety level 3 laboratories. Therefore, many clinicians wanted to know the cutoff value of Ct values for viable viruses. Furthermore, there is a strong need for a simple and rapid test which can be performed in minutes to determine the quarantine along with the increased number of patients during a pandemic. To address this need, the first antigen-detecting rapid diagnostic test (Ag-RDT) was authorized by the United States Food and Drug Administration in May of 2020 [13] and by the Ministry of Food and Drug Safety in Korea in November of 2020 [14]. The Ag-RDT has the advantage of being able to be used at point-of-care settings; in terms of efficacy, it shows low sensitivity but high specificity [13,14,15]. Thus, negative results from Ag-RDT may need to be confirmed with RT-PCR. In this study, we investigated Ct values which could be correlated with viable virus in patients with negative results in Ag-RDT and positive results in RT-PCR. Furthermore, we outline the admission protocols of our hospital and investigate the demographics, laboratory, and radiologic findings of patients admitted to a COVID-19- dedicated hospital in Korea.

## 2. Materials and Methods

**Figure 1:**
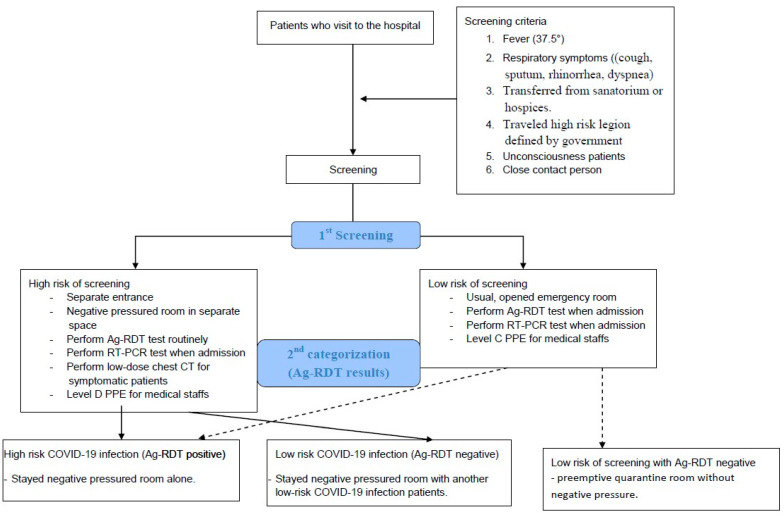
The admission protocol of our hospital.

This was a retrospective cohort study of 646 patients who were admitted to the hospital between 1 December 2020, and 28 February 2021, during the third wave of COVID-19 in Korea. Ag-RDT and RT-PCR tests were routinely performed on all patients who required admission, and low-dose chest computed tomography (LDCT) was performed on high-risk screening patients with associated symptoms. (Figure 1) We conducted the Standard Q COVID-19 Ag Test (SD Biosensor, Inc., Suwon, Korea) and Allplex™ SARS-CoV-2 Assay (Seegene, Seoul, Korea) according to the manufacturer’s instructions. Among the 646 patients, 335 patients with respiratory symptoms underwent LDCT. Ethical approval was obtained. (BIO-IRB 2021-002). 

Data, including comorbidity, patient’s symptoms, and laboratory tests including Ct values of RT-PCR test (nucleocapsid protein (N) gene, RNA-dependent RNA polymerase (RdRp) gene, and envelope protein (E) gene), were collected. The patients’ history of hypertension, ischemic cardiovascular disease, diabetes mellitus, renal insufficiency, and neurovascular diseases and asthma, were reviewed to assess the presence of comorbidities. The patient’s symptoms were evaluated, including fever, cough, sputum, rhinorrhea, dyspnea, myalgia, nausea, vomiting, and headache.

### 2.1. Protocols for Admission to Our Hospital

The overall protocol of our hospital is shown in Figure 1.

(1) Emergency department

First, patients who visited the emergency department were categorized into two groups: high risk of screening and low risk of screening. Patients at high risk of screening were defined as those with fever (≥37.5°), respiratory symptoms (cough, sputum, rhinorrhea, and dyspnea), patients transferred from sanatoriums or nursing hospitals, patients who traveled from high-risk regions defined by the government, unconscious patients, and close contact cases. The high risk of screening patients were kept in the negative pressured room, entering through a separate entrance. The medical staff who worked in the negative pressure room wore Level D personal protective equipment (PPE). Patients at high risk of screening were categorized again into two groups according to the results of the Ag-RDT test: high risk of COVID-19 infection and low risk of COVID-19 infection. A high risk of COVID-19 infection was defined as a positive result in Ag-RDT and stayed alone in the negative pressured room. Furthermore, all symptomatic patients underwent LDCT in a separate room. The low-risk COVID-19 infection patients were maintained under cohort quarantine in the negative pressured room with another low-risk COVID-19 patient (up to two people in a single negative pressured room). The patients were released from quarantine if their RT-PCR results were negative.

Patients categorized as having a low risk of screening stayed in the open emergency room. However, the medical staff wore Level C PPE. When admitted, the Ag-RDT test and RT-PCR were also performed. If the Ag-RDT test was positive, the patients stayed in the negative pressured room until the results of RT-PCR could be reported, similar to the process for high-risk COVID-19 patients. Patients with negative Ag-RDT test stayed in the quarantine room without negative pressure until the RT-PCR results could be reported.

(2) Out-patient department

Patients who visited the outpatient department obtained an identification card with the barcode of our hospital; this personal barcode was required for patients to enter the hospital along with the measuring the body temperature by a non-contact thermometer. If the patient’s body temperature was measured under 37.5 °C, they finally entered the hospital with an identification marker attached to the wrist, indicating that the patient had entered the gate appropriately. If the patients required immediate hospitalization, Ag-RDT and RT-PCR were performed with the same protocol of the emergency room. Patients who did not need to be immediately hospitalized underwent RT-PCR testing before admission within 3 days.

(3) Operation room

All emergent operations were performed in the negative pressured operation room by healthcare professionals wearing an N95 mask and goggle/facial shield with an antiseptic surgical gown after obtaining Ag-RDT results. A powered air-purifying respirator (PAPR) was used during the operation for high-risk patients. Non-emergent operations were delayed until RT-PCR results were obtained. 

### 2.2. Radiologic Evaluations

**Figure 2:**
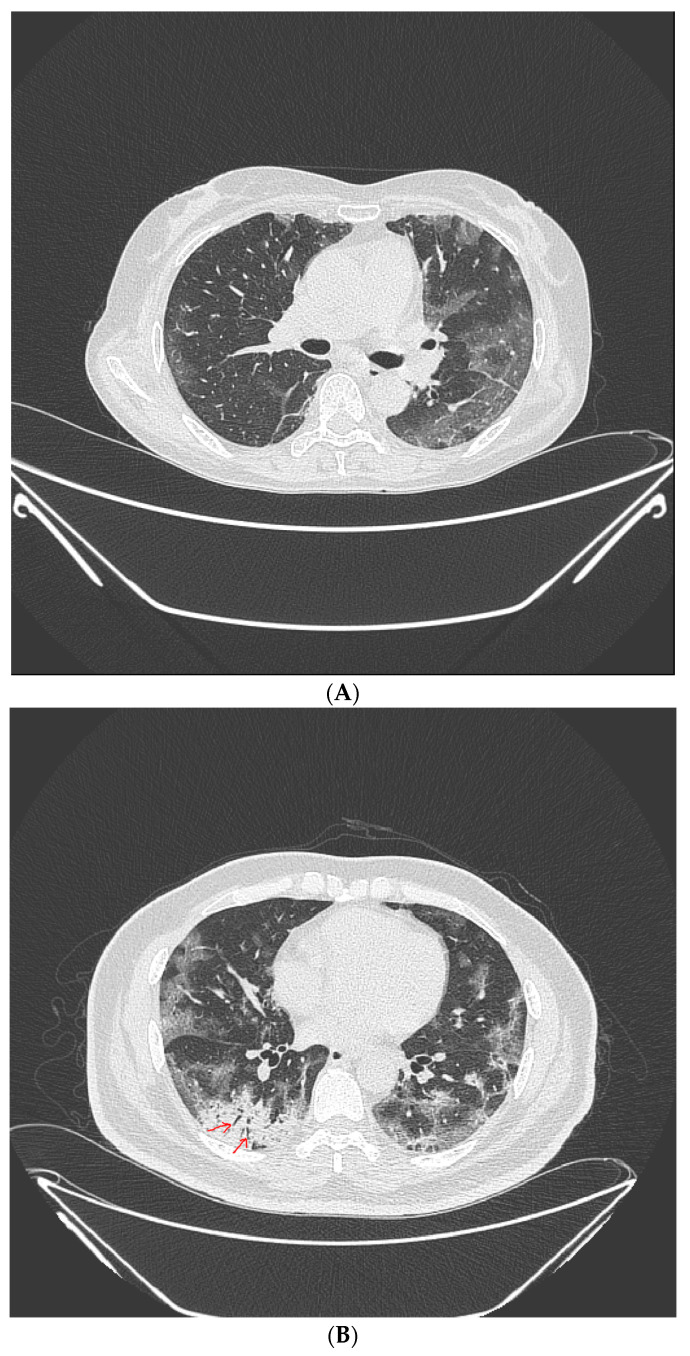

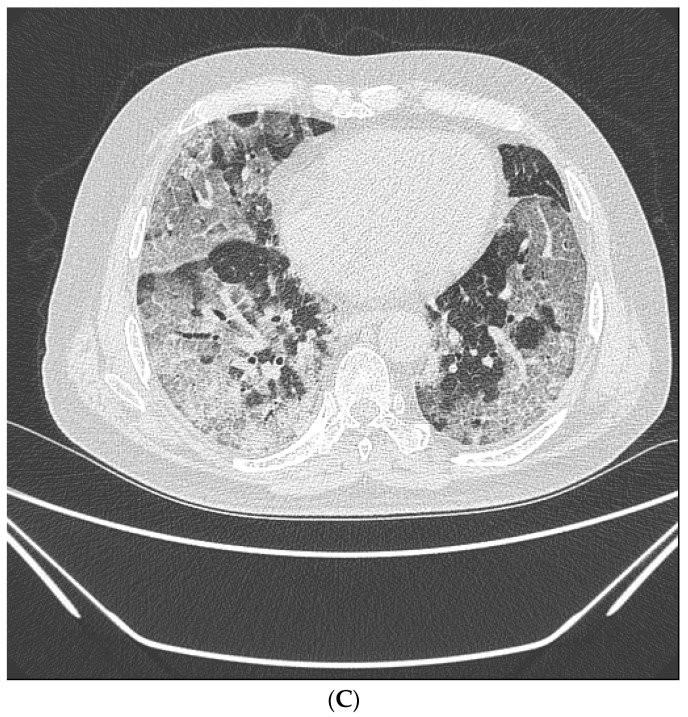
Typical findings for COVID-19 on CT scans. (**A**) Ground-glass opacity shows as a modest increase in lung attenuation on lung window CT images, not obscuring the pulmonary vessels. (**B**) Consolidation appears as high-density patchy opacities that obscure the margins of vessels and airway walls, inside which air bronchogram (arrow) could be observed. (**C**) Crazy-paving pattern appears as thickened interlobular septa and intralobular lines superimposed on a background of ground-glass opacity.

LDCT were obtained on symptomatic patients at high risk of screening with a 64-section scanner (Brilliance CT, Philips Healthcare) [16]. One radiologist (over 15 years of experience) and one pulmonology specialist (over 8 years of experience) retrospectively reviewed all LDCT images. Lobar distribution was assessed by observing the number of involved lobes, laterality, cephalocaudal distribution, and axial distribution [17]. In terms of pattern, ground glass opacity (GGO), consolidation, and crazy-paving pattern was assessed according to the definitions based on the Fleischner Society Nomenclature Committee recommendations [17,18]. (Figure 2A–C) Observation of the following characteristics was considered a typical finding for COVID-19; (1) peripheral, bilateral, GGO with or without consolidation or visible intralobular lines (“crazy-paving”), (2) Multifocal GGO of rounded morphology with or without consolidation or visible intralobular lines (“crazy-paving”), (3) Reverse halo sign or other findings of organizing pneumonia [18].

### 2.3. Statistical Analysis

The results were analyzed using the statistical software SPSS 19.0 IBM Corp. (Armonk, NY, USA). The diagnostic values may change, according to the presence of COVID-associated symptoms, and subgroup analysis for diagnostic values of symptomatic COVID patients was performed. The associated symptoms were defined as fever, cough, sputum, dyspnea, myalgia, rhinorrhea, or headache. Categorical variables were analyzed with the chi-squared test, and continuous variables were analyzed using the Mann–Whitney U-test, independent *t*-test, or Wilcoxon signed-rank test, according to the normality by the Shapiro–Wilks test. A receiver-operating characteristic (ROC) curve analysis was performed to determine the appropriate cut-off Ct values of RT-PCR for a positive Ag-RDT test. A post-hoc power analysis for significant AUC values was performed for Ct values of RT-PCR. We accepted an α error of 5% and β error of 20% to detect any significant differences. Based on these calculations, the required sample size for the N gene level was a minimum of 11 in total (7 cases of positive/4 cases of negative at least), with a null hypothesis value of 0.5, which means a robust result. 

## 3. Results

### 3.1. Demographics and Diagnostic Values of Ag-RDT and LDCT for High Risk of Screening Patients

The demographics of the 646 admitted patients are summarized in Table 1. Among the 646 patients who underwent Ag-RDT and RT-PCR, 4.8% (31/646) were positive by Ag-RDT and 6.3% (41/646) by RT-PCR tests. Thus, the overall Ag-RDT for COVID-19 had a sensitivity of 68.3%, specificity of 99.5%, positive predictive value (PPV) of 90.3%, and negative predictive value (NPV) of 97.9%. Among the three patients with positive Ag-RDT but negative RT-PCR, two patients did not have any symptoms. 

Positive RT-PCR results were most commonly found in patients with fever (*p* < 0.001); 41.5% of RT-PCR positive patients had a cough (odds ratio (OR) = 32.26; 95% confidence interval (CI) = 14.07–73.94), 24.4% of patients had sputum (OR = 6.41; 95% CI = 2.87–14.32), 26.8% of patients had myalgia (OR = 24.28; 95% CI = 9.35–63.05), 41.5% of patients had dyspnea (OR = 4.0; 95% CI = 2.07–7.74), and 60.9% of patients had headache (OR = 3.87; 95% CI = 2.02–7.43). There was no difference in the presence of comorbidities between the groups (all *p* > 0.05). 

For the subgroup analysis of 355 symptomatic patients with LDCT, the diagnostic value of Ag-RDT for COVID-19 had a sensitivity of 73.0%, a specificity of 98.8%, a PPV of 87.1%, and a NPV of 96.9%. The diagnostic value of LDCT for typical COVID-19 findings had a sensitivity of 48.6%, a specificity of 98.7%, a PPV of 81.8%, and an NPV of 94.3%. When the evaluations of Ag-RDT and LDCT were combined with the observation of typical COVID-19 findings, the diagnostic values had a sensitivity of 91.8%, specificity of 98.7%, PPV of 89.5%, and NPV of 99.1%. Overall diagnostic values of Ag-RDT, LDCT and combined Ag-RDT and LDCT were summarized in Table 2. 

### 3.2. Results of Laboratory Test including Ct Values of RT-PCR

The overall laboratory tests are summarized in Table 3. The total white blood cell (WBC) count was lower in the RT-PCR-positive group (*p* = 0.010), but there was no significant difference in the differential counts (*p* > 0.05). When comparing the Ct values of RT-PCR according to the results of Ag-RDT results, Ct values were low for the RdRp, E, and N genes from patients with positive Ag-RDT, but a significant difference was observed only in the N gene (*p* < 0.001, Table 3). 

### 3.3. Results of Ag-RDT Compared to Ct Values of Each Gene through RT-PCR

**Figure 3:**
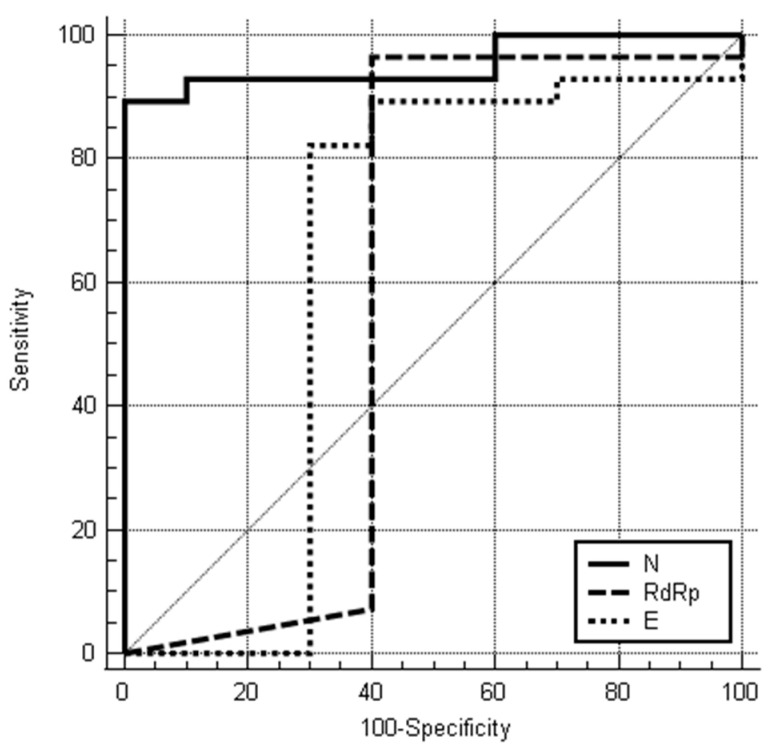
The ROC curves of Ct value for each gene through RT-PCR test. Abbreviations: ROC, receiver operating characteristic; Ct, cycle threshold; RT-PCR, reverse transcription polymerase chain reaction.

In ROC curve analysis for positive Ag-RDT compared to the Ct values, the significant AUC was set as 0.954 (95% CI: 0.831–0.995, *p* < 0.001) for the N gene with ≤ 25.67 of cut-off Ct value for a positive Ag-RDT (sensitivity: 89.3%, specificity: 100%). (Table 4 and Figure 3) The highest Ct values of each gene with positive Ag-RDT were found as 36.28 for E gene, 32.39 for RdRp gene and 34.99 for N gene.

### 3.4. Radiologic Findings on LDCT

The findings of LDCT in 355 symptomatic patients are summarized in Table 3. The κ values for inter- and intraobserver reliability ranged from 0.939 to 0.995, indicating excellent agreement (*p* < 0.001). Of the symptomatic patients, 25.9% (*n* = 92) had any type of pneumonia. Of these, 22 patients were diagnosed with COVID-19 using RT-PCR. Of patients diagnosed with pneumonia through LDCT, typical COVID-19 findings were found significantly in the positive RT-PCR group (*p* < 0.001). Furthermore, the density on LDCT was significantly different between the COVID-19 group confirmed by RT-PCR and the non-COVID-19 group (*p* < 0.001). The GGO pattern was found more frequently in the positive RT-PCR group (50.0%), whereas consolidation was found more frequently in the negative RT-PCR group (54.3%).

## 4. Discussion

The most important finding of this study was that the Ag-RDT has the potential to evaluate COVID-19 infection before obtaining RT-PCR results along with the screening protocol of this hospital. Furthermore, the diagnostic values of combined use of Ag-RDT and LDCT were found to be higher for the high-risk COVID-19 patients with symptoms than for those with Ag-RDT alone. Since RT-PCR might not be as fast as Ag-RDT or LDCT in all hospitals, these combined evaluations could be helpful in deciding whether strict quarantine would be necessary for admitted patients. However, due to the radiation hazards of LDCT, routine evaluations should not be considered for asymptomatic patients or low risk of screening patients.

In previous studies, the correlation between Ct value and sample infectivity through cell culture was strongly observed [8,9,10,11,12,19], but it is difficult to perform in clinical setting. Furthermore, using the RT-PCR system requires several hours to perform the RNA extraction and allow the RT-PCR to run; thus, Ag-RDT is useful in point-of-care settings during pandemic crises [13,19]. However, although Ag-RDTs have high specificity, they have low sensitivity, meaning that clinicians can obtain false negative results without confirmatory RT-PCR test [19]. In emergent or outbreak situations, point-of-care testing with a more theoretical basis is helpful. Many studies comparing Ct values with virus viability in cell culture have been reported that cut off for viable viruses have a Ct value ranging from 25 to 30 [8,9,10,11,12]. Based on these previous studies, it is necessary to clarify Ag-RDT results compared with Ct value in order to treat non-COVID-19 patients [19]. In previous studies, the mean Ct values of Ag-RDT negative results were greater than 30, at which point viruses might have a low possibility of viability in cell culture [8,9,10,11,12,19,20]. In this study, the cut-off value of N gene in the RT-PCR test was 25.67 for the negative Ag-RDT test (Table 4), and the Ct value of N gene was 32.43 for the negative Ag-RDT. Thus, the negative Ag-RDT test might reveal a high Ct value in the N gene—25.67 at least—as a low viable virus in cell culture. In brief, we could consider the use of Ag-RDT more actively in the field of care to categorize or screen patients as high- or low-risk, in conjunction with considering their symptoms or history.

In this study, the sensitivity of Ag-RDTs was 68.3%, but the specificity for high-risk patients was shown higher, as 99.5%, respectively. Moreover, among symptomatic patients, the diagnostic value of Ag-RDT for COVID-19 was shown to be higher, with a sensitivity of 73.0% and a specificity of 98.8%. However, when combining the evaluations of Ag-RDT and LDCT with typical COVID-19 findings, the diagnostic values had a sensitivity of 91.8% and specificity of 98.7%, although the diagnostic values of LDCT alone were found to be lower than 50% in sensitivity. If Ag-RDT is negative but RT-PCR results are pending, LDCT could be selectively conducted to rule out COVID-19 infection in symptomatic patients in an emergency [16], with careful consideration of radiation hazards [21]. In this study, among the four patients of positive LDCT findings but negative RT-PCR at the time of hospital visit, one patient turned out to be positive in RC-PCT test after 3 days from initial evaluations including Ag-RDT and LDCT. 

According to our hospital protocol, there were no patients or medical staff who became infected with COVID-19 in the hospital; thus, our procedure could be implemented to increase safety for healthcare workers and facilitate the running of the facility. Moreover, during surgery, including emergency surgeries of orthopedics and other departments, there was no single case that violated the national guidelines that needed to shut down the facility. Furthermore, this protocol would be helpful not only for infection control, but also for scheduling elective surgery for essential treatment. 

There have been many discussions and reviews investigating the viability of ‘elective’ surgery as well as safe emergency surgery [22,23,24,25]. Most of the previous studies reported and emphasized the necessity for screening protocol, guidelines, or considerations before starting surgery, and a lack of experience reporting according to the structured guidelines was perceived [22,23,24,25,26]. Moreover, this hospital is designated as a COVID-19 specialized hospital by the Ministry of Health and Welfare in Korea; thus, sharing our experiences would be helpful to proceed with hospitalization and surgeries, including elective or emergency. The guidelines of our hospital could play an appropriate role for the community on outpatients, inpatients, and surgeries, as well as COVID-19 screening, treatment, and quarantine. 

Our study had some limitations. First, because of the low prevalence of COVID-19 in Korea, the sample size was small. A larger sample size study is needed to reveal the accuracy of Ag-RDT compared to the Ct values of RT-PCR. Second, this study was conducted retrospectively in a clinical setting. Thus, we did not check for viable virus through cell culture, and only quoted the viable Ct values from other studies. Third, there may be differences among the diagnostic kits of different companies or countries. Furthermore, there also may be differences in patient’s general condition, comorbidities, ages, etc. 

## 5. Conclusions

Appropriate patient care was possible by definitive triage of the area, according to the symptoms and through the use of diagnostic tests. Screening protocols, including the Ag-RDT test and LDCT, could be helpful in emergency point-of-care settings if the RT-PCR test is pending.

## Figures and Tables

**Table 1:** Results of demographics, antigen-detecting rapid diagnostic test in admitted patients.

	RT-PCR Positive, *n* = 41	RT-PCR Negative, *n* = 605	*p*-Value
**Ag-RDT**			<0.001
Positive	28	3	
Negative	13	602	
**Sex**			0.868
Male	21	318	
Female	20	287	
**Age (mean** **±** **SD)**	70.1 ± 13.1	63.4 ± 19.9	0.033
**Body mass index**	22.4 ± 3.1	23.4 ± 4.0	0.122
**Symptoms**			
Fever	37.7 ± 0.8	36.8 ± 0.7	<0.001
Cough	17	13	<0.001
Sputum	10	29	<0.001
Myalgia	11	9	<0.001
Rhinorrhea	0	4	1.000
Dyspnea	17	91	<0.001
Nausea/Vomiting	3	57	0.206
Headache	25	174	<0.001
**Comorbidities**			
Diabetes mellitus	11	148	0.734
Hypertension	24	262	0.057
Ischemic heart disease	3	32	0.579
Renal insufficiency	1	20	0.762
Neurological disease	3	32	0.579
Asthma	1	15	1.000

Abbreviation: RT-PCR; reverse transcription polymerase chain reaction, Ag-RDT; antigen-detecting rapid diagnostic test, SD; standard deviation.

**Table 2:** Overall diagnostic values of Ag-RDT and LDCT.

	Ag-RDT		LDCT	Ag-RDT + LDCT
	All Patients	Symptomatic Patients	Symptomatic Patients	Symptomatic Patients
Sensitivity, %	68.3	73.0	48.6	91.8
Specificity, %	99.5	98.8	98.7	98.7
Positive Predictive value, %	90.3	87.1	81.8	89.5
Negative Predictive value, %	97.9	96.9	94.3	99.1

Abbreviation: Ag-RDT; antigen-detecting rapid diagnostic test, LD CT; low-dose chest computed tomography.

**Table 3:** Laboratory and radiologic findings in admitted patients.

	RT-PCR Positive, *n* = 41 (%)	RT-PCR Negative, *n* = 605 (%)	*p*-Value
**WBC, 10^9^/L**	7.83 ± 5.84	9.84 ± 4.74	0.010
**Neutrophil (%)**	71.2 ± 15.1	73.4 ± 14.4	0.344
**Lymphocyte (%)**	19.1 ± 11.9	18.3 ± 12.2	0.670
**Platelet, 10^9^/L**	217.2 ± 84.4	237.4 ± 82.7	0.131
**CRP, mg/L**	5.78 ± 7.52	3.76 ± 6.64	0.061
**Ct values of RT-PCR, (mean** **±** **standard deviations(** **SD** **)** **)**			
RdRp gene	17.95 ± 10.66		
E gene	20.17 ± 9.87		
N gene	20.54 ± 10.47		
**Ct values of RT-PCR according to the Ag-RDT results, (mean** **±** **SD)**			
RdRp gene			0.668
Positive Ag-RDT, *n* = 28	17.30 ± 7.23		
Negative Ag-RDT, *n* = 13	19.35 ± 16.08		
E gene			0.263
Positive Ag-RDT, *n*= 28	18.68 ± 7.31		
Negative Ag-RDT, *n*= 13	23.39 ± 13.71		
N gene			<0.001
Positive Ag-RDT, *n* = 28	16.29 ± 8.60		
Negative Ag-RDT, *n* = 13	32.43 ± 4.01		
**Chest CT findings, *n* = 355**	*n* = 37	*n* = 318	
No pneumonia	15 (40.5)	248 (78.0)	<0.001
Any type of pneumonia	22 (59.5)	70 (22.0)	
Any COVID-19 typical findings	18	4	<0.001
**Involvement of the lesion**			0.299
Single lobe	4	11	
Multilobular	18	59	
Numbers of involved lobe	3.77 ± 1.72	3.43 ± 1.47	0.370
**Laterality**			0.952
Rt	4	13	
Lt	2	5	
Both	16	52	
Cephalocaudal distribution			0.407
Upper	1	1	
Middle	1	0	
Lower	5	20	
Diffuse	15	49	
**Axial distribution** **^†^**			0.115
Central	1	3	
Peripheral	13	26	
Diffuse	8	41	
**Density**			<0.001
GGO	11 (50.0)	13 (18.6)	
Crazy-paving pattern	3 (13.6)	2 (2.9)	
Mixed GGO and consolidation	7 (31.8)	17 (24.3)	
Consolidation	1 (4.5)	38 (54.3)	

Abbreviation: RT-PCR; reverse transcription polymerase chain reaction, WBC; white blood cell, CRP; C-reactive protein, Ct; cycle threshold, Ag-RDT; antigen-detecting rapid diagnostic test, RdRp; RNA-dependent RNA polymerase gene, E gene; envelope protein gene, N gene; nucleocapsid protein gene, CT; computed tomography, GGO; ground glass opacity/^†^ For the axial distribution, the outer 1/3 of the lung field was defined as peripheral distribution, whereas the remainder was defined as central distribution.

**Table 4:** Criterion values and coordinates of the receiver operating characteristic curves for positive Ag-RDT test with Ct levels of RT-PCR test.

	AUC	95%CI of AUC	Cut-off	Sensitivity, %	Specificity, %	*p*-Value
**RdRp**	0.610	0.445–0.758	≤25.16	96.4	61.5	0.408
**E gene**	0.695	0.532–0.829	≤24.2	82.1	76.9	0.096
**N gene**	0.954	0.831–0.995	≤25.67	89.3	100	<0.001

Abbreviation: RT-PCR; reverse transcription polymerase chain reaction, AUC; area under the curve, CI; confidence interval, RdRp; RNA-dependent RNA polymerase gene, E gene; envelope protein gene, N gene; nucleocapsid protein gene.

## Data Availability

The data that support the findings of this study are available from the corresponding author upon reasonable request.
